# Dietary Patterns in European and Brazilian Adolescents: Comparisons and Associations with Socioeconomic Factors

**DOI:** 10.3390/nu10010057

**Published:** 2018-01-09

**Authors:** Camila Aparecida Borges, Betzabeth Slater, Alba Maria Santaliestra-Pasías, Theodora Mouratidou, Inge Huybrechts, Kurt Widhalm, Frédéric Gottrand, Yannis Manios, David Jimenez-Pavón, Jara Valtueña, Cinzia Le Donne, Ascensión Marcos, Dénes Molnar, Manuel J. Castillo, Stefaan De Henauw, Luis A. Moreno

**Affiliations:** 1 School of Public Health, Department of Nutrition, University of Sao Paulo Avenue Dr. Arnaldo 715, Sao Paulo 01246-904, Brazil; slaterbetzy@gmail.com; 2 GENUD (Growth, Exercise, Nutrition and Development) Research Group, Universidad de Zaragoza, Agroalimentary Institute of Aragon (IA2), Institute of Health Research of Aragon (IIS Aragon), Center for Biomedical Research Network Pathophysiology of Obesity and Nutrition (CIBERObn), 50009 Zaragoza, Spain; albasant@unizar.es (A.M.S.-P.); theodora@unizar.es (T.M.); lmoreno@unizar.es (L.A.M.); 3 International Agency for Research on Cancer, 150 Cours Albert Thomas, 69372 Lyon CEDEX 08, France; inge.huybrechts@ugent.be; 4 Medical University of Vienna, Waehringer Guertel 18-20, A-1090 Vienna, Austria; kurt.widhalm@meduniwien.ac.at; 5 Inserm U955, IFR 114/IMPRT, Faculty of Medicine, University Lille 2, F-59037 Lille, France; frederic.gottrand@chru-lille.fr; 6 Department of Nutrition and Dietetics, Harokopio University, 70 El Venizelou Ave, 176 71 Athens, Greece; manios.helena@hua.gr; 7 MOVE-IT Research Group, Department of Physical Education, Faculty of Education Sciences, University of Cádiz, 11519 Cádiz, Spain; david.jimenez@uca.es; 8 ImFINE Research Group, Department of Health and Human Performance, Facultad de Ciencias de la Actividad Física y del Deporte (INEF), Universidad Politécnica de Madrid, C/Martín Fierro, 7, 28040 Madrid, Spain; jara.valtuena@upm.es; 9 Council for Agricultural Research and Economics, Research Centre for Food and Nutrition, Via Ardeatina, 546, 00178 Rome, Italy; cinzia.ledonne@crea.gov.it; 10 Institute of Food Science, Technology and Nutrition (ICTAN), Spanish National Research Council (CSIC), C/Jose Antonio Novais 10, 28040 Madrid, Spain; amarcos@ictan.csic.es; 11 Department of Pediatrics, University of Pécs, Medical School, József A. u. 7., 7623 Pécs, Hungary; denes.molnar@aok.pte.hu; 12 Department of Medical Physiology, Faculty of Medicine, University of Granada, Avenida Madrid, 12, 18012 Granada, Spain; mcgarzon@ugr.es; 13 Faculty of Medicine and Health Sciences, Department of Public Health, Ghent University, 9000 Ghent, Belgium; stefaan.dehenauw@ugent.be

**Keywords:** dietary patterns, adolescents, socioeconomic status, factor analysis

## Abstract

Associations between dietary patterns (DP) and socioeconomic factors have been little explored in adolescents. The aim of this study was to identify DP in European and Brazilian adolescents and to investigate their associations with a range of socioeconomic indicators. Adolescents from the HELENA-study and the Household Budget Survey were analyzed. Factor analysis was used to obtain DP. Linear regression was used to examine the association between DP and SES. In Europeans, the Western DP was associated with low education of the mother, high socioeconomic status (boys), older age (boys), and living in cities of the Northern Europe; in Brazilians, the Western DP was associated with high secondary education of the mother, high socioeconomic status and living in Southern areas of the country. The Traditional European DP, in both genders, was associated with high secondary education of the mother and inversely associated with a high socioeconomic status; the Traditional Brazilian DP, was associated with university level education of the mother and older age (boys). The association between DP and socioeconomic factors is relevant for the understanding of food-related practices and highlight the importance of performing a complete assessment of the socioeconomic influence in adolescent’s DP from developed and developing countries.

## 1. Introduction

Non-communicable diseases are currently the main cause of mortality and morbidity in both developed and developing countries [1], with nutrition-related factors being one of the main determinants [2]. Some argue that addressing diet-disease associations on the basis of individual foods and/or nutrients might not be the best approach, since foods and nutrients as a whole form part of an individual’s diet [3]. Thus, studying dietary patterns (DP) has been suggested as a complementary method to investigate such associations [4]. For instance, a DP combining foods that are rich in fats, sugars, and salt, known as Western DP, has been associated with increased risk of cardiovascular diseases [5], hypertension [6], type 2 diabetes [7], osteoporosis [8], some types of cancer [9], and obesity [10,11].

DP established in childhood are consolidated in adolescence and will remain for life [12]. Studies from around the world indicate that DP of adolescents are characterized by limited variety of foods and a high intake of snacks and sugar-sweetened beverages [13,14,15]. During adolescence, results from different studies also showed that some DP, defined as Western DP, Unhealthy DP, or Processed DP, were associated with increased obesity risk [14,16,17].

Associations between DP and socioeconomic factors (SES) have been well explored in adult population-based studies. However, there is a lack of such studies in adolescents [18]. SES is one of the main determinants of human health [1,19,20]. SES, such as total family income, parental education, parental occupation, and family welfare determines food choices and access to ‘healthy’ and ‘unhealthy’ foods [19]. In studies regarding DP in adolescents, SES was identified as one of the main determinants of a healthy diet [14,17,21,22,23]; however, its influence is still little explored. DP that include a variety of healthy foods were directly or inversely associated to SES, and this association depended on the social context of each country [18,21,24,25]. Assessing the relationship between DP and SES in adolescents from a diverse set of countries could support the development of appropriate food and nutrition policies and regulations for this stage of life. Therefore, the aim of this study was to identify DP in European and Brazilian adolescents and to explore their associations with a range of socioeconomic indicators.

## 2. Materials and Methods

### 2.1. Sample

This study analyzed two surveys conducted among European and Brazilian adolescents. The Healthy Lifestyle in Europe by Nutrition in Adolescence (HELENA-study) is a multicenter study focusing on lifestyle and nutrition of adolescents from 10 European cities (Athens, Heraklion, Dortmund, Ghent, Lille, Pecs, Rome, Stockholm, Vienna, and Zaragoza) conducted between 2006 and 2007. To ensure heterogeneity of social background, participants were recruited at random. Up to three classes from two grades were selected per school. More details on operational and sampling procedures have been previously published [26]. In total, 3528 (46.9% boys) adolescents met the HELENA inclusion criteria: being 12.5–17.5 years old, not participating simultaneously in a clinical trial and being free of any acute infection lasting less than one week prior to the inclusion. For logistical reasons, data from Heraklion and Pecs were not included in the dietary intake analysis (7% of the total sample). Extra inclusion criteria for the purpose of the current analysis included: having complete SES variables and having provided two 24-h dietary recalls (24 H-DR), resulting in 2330 adolescents. The study was approved by the Research Ethics Committees of each city involved (Athens and Heraklion (Greece): approved by the Ministry of Education, Research and Religious Affairs (protocol number 79162); Dortmund (Germany): approved by the University Klinik Bonn (protocol number 91209-07); Ghent (Belgium): approved by the University of Ghent (protocol number 007034); Lille (France): approved by the Ethics Committee for the Protection of Persons Participating in Biomedical Research (number CP06/12); Pecs (Hungary): approved by Regional Research Ethics Committee of the Medical Center, Pécs; Rome (Italy): approved by the Ethics Committee of the University of Naples Federico II-Ethics Committee for Biomedical Activities (protocol number C.E. n 95/06); Stockholm (Sweden): approved by the Regional Ethics Committee-EPN (protocol number 2007/2-17); Vienna (Austria): approved by the University of Vienna (protocol number 535/2005); Zaragoza (Spain): approved by the Ethics Committee of the government of Aragon-department of health and consumption (protocol number 06/01). Written informed consent was obtained from the parents of the adolescents and the adolescents themselves [27].

The Brazilian National Dietary Survey (NDS) was carried out between 2008–2009 and embedded for the first time in the Household Budget Survey (HBS), a national investigation conducted by the Brazilian Institute of Geography and Statistics (IBGE). The sample for the Brazilian HBS was selected using a two-stage cluster sampling design. In the first stage, the primary sampling units (census tracts) were selected by systematic sampling with probability proportional to the number of households. Census tracts were stratified to include all Brazilian regions, including both urban and rural areas, and different socio-economic categories. In the second stage, households were selected by simple random sampling. Individuals that were aged 10 years or older (*n* = 34,003) living in the selected households were included in the NDS. More details on operational and sampling procedures have been previously published [28]. For the purposes of this study and to ensure comparability of results between European and Brazilian adolescents, only Brazilian adolescents (12.5–17.5 years), with complete SES information, with two dietary records and living in urban areas were included, resulting in 3194 adolescents. The NDS study was approved by the local ethics committee (CAAE 0011.0.259.000-11). The present study has been approved by the local ethical committee (CAAE 0129.0.207.000-11; protocol number: 2315) of the School of Public Health/Sao Paulo, Brazil.

### 2.2. Dietary Intake Assessment

In European adolescents, dietary intake was assessed using the self-administered, computerized 24 H-DR HELENA-Dietary Assessment Tool (DIAT) based on the Young Adolescents’ Nutrition Assessment software (YANA-C). The validity of the tool in European adolescents for all nutrients and energy intakes ranged from 0.86–0.91 [29]. The adolescents completed two 24 H-DR [30] during school period and within a time span of two weeks; on both occasions, trained dietitians were present. The Multiple Source Method [31] was used to calculate usual energy intake removing the effect of day-to-day within-person variability and random error in the recalls. The 43 food groups included in the HELENA-DIAT list [29] were aggregated into 28 food groups, according to their nutritional values, and were thereafter included in subsequent analysis (Table A1).

In Brazilian adolescents, dietary intake was assessed using dietary records completed during two non-consecutive predetermined days. All of the individuals reported all foods and beverages consumed, including the amount consumed in household measurement units, times of every intake occasion, recipe, and eating occasion. When the individual was unable to fill in the dietary record, this was completed with the help of another household member or a person that was appointed by the individual. The information on the dietary records was reviewed during a personal interview in the participant’s home. Data from the dietary records were stored using a software developed specifically for this research [28]. Partial dietary intake analysis was performed during data collection, as the main quality control strategy [32]. For example: frequency response, mean consumption of individual items in the first and second day, and not registered code items among others, were checked. Details on the pre-test, training, validation of the data collection, imputation process and data entry have been previously published [33,34]. The participants mentioned 1971 food items that, in this study, were organized into 28 groups, according to their nutritional values (Appendix A). The Multiple Source Method [31] was used to calculate usual energy intake removing the effect of day-to-day within-person variability and random error in the records.

### 2.3. Socioeconomic Factors

The Family Affluence Scale (FAS) was used in European data. This scale consists of four items: household availability of cars, personal computers, internet, and number of bedrooms [18]. The final score for each individual was stratified into three categories (low, medium, high). We also used other variables: education of father and mother (low education, high secondary education, and university degree), occupation of father and mother (low occupation, medium occupation, high occupation, and undefined class), number of siblings (0, 1, >2), number of persons living in the household (2–3, 4, 5, >6), living with parents (yes, no), and geographical region (North: Dortmund in Germany, Ghent in Belgium, Lille in France, Stockholm in Sweden, Vienna in Austria and South: Athens in Greece, Rome in Italy, and Zaragoza in Spain) [35].

In Brazilian adolescents, we used the total family income, education of the mother (low education, high secondary education and university degree), number of persons living in a household (2–3, 4, 5, >6), and geographical region (North: North, Northeast areas and South: South, Southeast, Midwest areas). 

### 2.4. Statistical Analysis

All of the analyses were gender-specific due to significant differences in SES and DP for boys and girls. According to the nature of the studied variables, analysis of variance was used to compare gender-specific sample characteristics. Factorial analysis (based on principal–component factor) with varimax rotation was used to obtain dietary patterns [36]. This technique is often used in data reduction to identify a small number of factors that explain most of the variance observed in a much larger number of variables by defining highly interrelated sets. Each DP obtained represents a linear combination of all food groups, which are weighted by their factor loading. Those with an absolute value >0.3 were considered important contributors to each DP [3]. In the current analysis, positive factor loadings (>0.30) indicate positive correlations between the food group and all of the factors included within the corresponding DP, and negative factor loadings (>−0.30) indicate negative correlations with the corresponding DP. The following criteria were used when deciding the number of factors to be retained: eigenvalue > 1, the screeplot (a graphical presentation of eigenvalues) and the interpretability of each component [37]. The first DP explains as much inter-individual variation of the foods as possible, the next DP explains as much of the remaining variation as possible, and so on. Each subject receives a score for each DP, with a higher score indicating a higher adherence to the respective pattern. The factor scores for each adolescent were used in subsequent analyses. Linear regression model examined the association between DP scores (dependent variable) and socioeconomic indicators. These associations were expressed as unstandardized regression β-coefficients (95% confidence interval, CI), indicating the change in the score of the DP in relation to the socioeconomic variables. All of the analyses were conducted in *software STATA* version 12. *p* values < 0.05 were considered statistically significant. 

## 3. Results

A total of 5407 adolescents were included in this study. 2213 of those (46% boys, mean age of 14.9 years (95% CI: 14.9, 15.1)) came from the HELENA study, and 3194 of those (51% boys, mean age 14.6 years (95% CI: 14.4, 14.7)) came from the HBS-Brazil. Table 1 shows the descriptive analysis of the studied variables stratified by gender, in both studies. In European adolescents, gender differences were found in the occupation level of the father (*p* < 0.05). In Brazilian adolescents, we identified gender differences in socioeconomic status (based on total family income) (*p* < 0.05) (Table 1).

Three DP were identified for European boys (21% total variance explained) and four DP for girls (24% total variance explained). In boys, the identified DP were: (1) Western DP composed of bread and bread rolls, savory snacks, non-chocolate confectionery, chocolate, butter/animal fats and margarine, sauces, coffee and tea, sugar-sweetened beverages, and cheese; (2) Traditional European DP composed of bread and bread rolls, cereals, vegetable oils/nuts/seeds, pulses, vegetables (excluding potatoes), and cheese, and, finally; (3) Breakfast DP composed of breakfast cereals, fruits, milk and dairy products. In girls, the identified dietary patterns were: (1) Breakfast DP composed of breakfast cereals, bread and bread rolls, sugar/honey/syrup, butter/animal fats and margarine, fruits and coffee/tea; (2) Western DP composed of sweet bakery products, savory snacks, non-chocolate confectionery, chocolate, sugar-sweetened beverages, and alcoholic beverages; (3) Traditional European DP composed of vegetable oils/nuts/seeds, pulses, vegetables (excluding potatoes), and eggs; and (4) Monotonous DP based on bread and rolls, cereals and cheese (Table 2).

Among Brazilian adolescents four DP were identified in boys (23% total variance explained) and four DP in girls (23% total variance explained). In boys, the identified DP were the following: (1) Traditional Brazilian DP composed of cereals, pulses, meat/chicken/sausages/ham bread and bread rolls, butter/animal fats and margarine, coffee/tea, and fruit/vegetable juices; (2) Western DP included savory snacks, non-chocolate confectionery, chocolate, and sugar-sweetened beverages; (3) Snacks DP composed of sweet bakery products, sauces, milk and dairy products; and (4) Healthy DP based on starchy roots and potatoes, fruits, meat/chicken/sausages/ham and fish. In girls, the identified dietary patterns were: (1) Western DP composed of sweet bakery products, savory snacks, non-chocolate confectionery, fruit/vegetable juices, Sugar-sweetened beverages, and cheese; (2) Breakfast DP was based on bread and bread rolls, sugar/honey/syrup, butter/animal fats, and margarine and coffee/tea; (3) Sweets and Fried Foods DP composed of breakfast cereals, vegetable oils/nuts/seeds, and fish; and (4) Traditional Brazilian DP consisted of cereals, pulses, fruits, fruit/vegetable juices, starchy roots/potatoes, and meat/chicken/sausages/ham (Table 3).

In European boys, we found more exposure to the *Western* DP (7.3% of variance explained) when compared to European girls (5.5% of variance explained) (Table 2). In Brazil, the Western DP was more prevalent in girls (6.4% of variance explained) than boys (6.2% of variance explained). The Traditional Brazilian DP explained more variance in Brazilian boys (6.8%) than in girls (5.2%) (Table 3).

In European boys, the Western DP was negatively associated with living in the South of Europe, and positively associated with: older age, high socioeconomic status, low education of the mother; the Traditional European DP was associated with older age, high secondary education of the mother, medium occupation of the father and living in the South of Europe; the Breakfast DP was associated with medium socioeconomic status, high secondary parental education or university degree, high parental occupation, and living with five or more persons in a household (Table 4).

In European girls positive associations were found between Breakfast DP and age between 15–17.49, high socioeconomic status, high parental occupation and negative associations when living in a country from Southern Europe; the Western DP was inversely associated with high levels of education and occupation of both parents and living in countries from Southern Europe; the Traditional European DP was associated with high secondary education of the mother, medium occupation level of the father, and living in countries from Southern Europe. It was also inversely associated with high socioeconomic status, high occupation level of the mother and living with six or more persons in a household; the Monotonous DP, was found to be associated with high socioeconomic status and high occupation level of the mother, but inversely associated with living in countries from the South of Europe (Table 4). 

In Brazilian boys, the Traditional DP was associated with older age, medium socioeconomic status and high secondary education level of the mother; the Western DP was associated with a high socioeconomic status, high secondary education of the mother and living in Southern areas; it was also inversely associated with living with >6 persons in a household; the *Healthy* DP was inversely associated with living in the South areas. No significant associations were found between the Snacks DP and SES variables (Table 5).

In Brazilian girls, the Western DP was associated with high socioeconomic status, high secondary, and university degree of the mother and living in the Southern areas; the Sweets and Fried Foods DP was associated with living with five persons in a household and inversely associated with living in the South regions; the Traditional Brazilian DP was associated with living in the Southern areas; it was also inversely associated with high socioeconomic status. No significant associations were found between the Breakfast DP and socioeconomic status among Brazilian girls (Table 5).

## 4. Discussion

The present study used data from the European HELENA study and the national Brazilian HBS study. Three similar DP were identified in both samples: Western, Traditional and Breakfast. The main findings of the study were the associations between these DP and socioeconomic and demographic indicators: age, education of the mother, parental occupation level, socioeconomic status, and South-North areas, which varied in its directions by geographical region (Europe vs. Brazil). To the author’s knowledge, this is the first study comparing these associations in Brazilian and European adolescents, when considering two representative samples with different geographical, cultural, and socioeconomic characteristics. 

The DP found in both studies exhibited differences and similarities in their food composition. For example: The Western DP in both studies comprised foods that were high in fat, sugar, salt, processed, not fresh and typical of a westernized lifestyle. Studies also identified this DP in European [16,38], Mexican [39], Japanese and Polish [40] adolescents. The *Western* DP is associated with an increased risk of non-communicable diseases in adolescence [3,16,25,40]; the Traditional European and Traditional Brazilian DP have in their composition typical food groups consumed in Europe (i.e., breads, vegetable oils, pulses, eggs, vegetables) and in Brazil (i.e., rice, beans, meat); the Breakfast DP in European adolescents comprised breakfast cereals and fruits and in Brazilians it was composed of breads, butter/margarine, coffee and sugar, all of these foods being part of a typical breakfast meal in both populations. 

Differences in the percentage of variance explained in each DP according to gender were also found. In girls, the percentage of variance explained by ‘unhealthy DP’ (based on the sum of the variances explained by Western and Monotonous DP in Europeans and by the Western and Sweets and Fried Foods DP in Brazilians) was higher than in boys. In respect of the differences in food consumption among both genders, Hiza, et al., 2013 [41] observed that adult women had better-quality diets than adult men and adolescents (both genders) had worst-quality diets than children and older adults. 

Positive associations between the older age and Western DP and negative associations between the same age category and Breakfast DP were found in European boys. These findings could suggest that the Western DP is acquired during adolescence while the Breakfast DP is dropped during this period in European boys. There are few studies analyzing the shift in DP among adolescents. In a longitudinal study, Borges, et al. observed that Brazilian adolescents moved from Healthy to Unhealthy DP [42]. The decrease of parental control and the availability of “pocket money” would allow for adolescents to decide what they want to eat, not always electing healthy foods [38]. In Brazilian adolescents, we only found association between Traditional DP and older age, which is the opposite of what we observed in European adolescents. 

Concerning the association between DP and maternal education, in European adolescents from both genders, low and high secondary education of the mother were associated with high scores of the Western and Traditional European DP, respectively; however, in Brazilian boys and girls, high secondary and university education of the mother were associated with high scores of the *Western* DP. A high maternal education was considered a positive influencing factor for healthy food intake, especially in childhood [21,43]. There are few studies in adolescents associating low education of the mother with DP. In UK adolescents [17], low education of the mother was associated with lower scores of ‘traditional/health conscious DP’. In Spanish adolescents [44], a low education of the mother was associated with the ‘snacky DP’ and negatively associated with the ‘healthy DP’. In Latin America, studies considered the education level of the head household member, preventing comparisons with the European studies.

High parental occupation level, living with the parents, having siblings, and living with five or more persons in a household was positively associated with the *Breakfast* DP in European adolescents. These associations suggested that DP of adolescents are associated with eating practices of all family members. Other studies observed associations with a healthy DP and parental occupation [23] and parental influences [12] among adolescents.

In European boys, high FAS was associated with the *Western* DP and inversely associated with a Traditional European DP in both genders, whereas, in Brazilian boys and girls, a high total family income was associated with the Western DP. Associations between high SES and *Western* DP showed unexpected results in European boys, since other studies have identified associations between low socioeconomic status and ‘unhealthy DP’ in developed countries [14,17,44,45,46]. There are a number of possible explanations for this finding. We used FAS as a proxy of socioeconomic status in European adolescents. FAS is an indicator assessing economic welfare of the family (ownership of electronic devices and cars), which is not necessarily related to income [18]. Adolescents from high socioeconomic status groups are used to receiving ‘pocket money’ to buy lunch and snacks. Foods rich in fat, sugar and salt are easily available for high socioeconomic status adolescents [47]. Finally, the Western DP found in two distinct geographical and socioeconomic populations are the result of common worldwide trends in DP over the last decades, in all social classes, with a shift from the Traditional and Healthy DP to the Western or Unhealthy DP [40,42,48,49,50]. 

In Europe [14,45] and US [51,52] studies also evaluated the associations between socioeconomic status and DP in adolescents. In Scottish adolescents [14], a low family income was associated with the ‘unhealthy DP’. In German adolescents [45], low family income was associated with the ‘western DP’. In our European boys, we found an opposite association. This discrepancy could be due to the different indicators used in the studies. In Latin America, we only found two similar studies that were conducted in Brazil. Rodrigues, et al. [51] found an association between a low family income and the Traditional DP (boys); however, in our study, the Traditional DP in Brazilian boys was associated with medium family income and no associations were observed in Brazilian girls. Marchioni, et al. [52] did not identified a Western DP, but a similar one, called ‘dual pattern’, which is characterized by dairy products, fruit, fruit juice, vegetables, processed meat, soft drinks, sweets, bread, and margarine; it was associated with a high total family income, as well as the Western DP identified in our Brazilian adolescents. 

We identified consistent associations between DP and geographical regions, both in Europe and Brazil. High Western DP scores were observed in countries of Northern Europe and in Southern urban areas of Brazil. Cruz J.A. [53] also showed that poor eating behaviors, illustrated by the practice of the snacking habits and eating out in fast food restaurants, were less frequent in adolescents from Southern countries when compared with adolescents from some Nordic countries. Samuelson G. [54] found a high prevalence of irregular meal patterns and skipping breakfast in adolescents from Northern Europe when compared with those from Southern Europe. In Brazil, Souza et al. [55] identified a DP based on rice, beans, coffee, bread, and beef in girls. This DP was also more prevalent in Southern areas. 

The importance of this study lies in the use of two representative samples of European and Brazilian adolescents and in the total number of adolescents included. Up to this point, few studies have used population-based surveys with large sample size to identify DP in adolescents [14,21]. Also, both studies (HELENA and HBS) used consolidated methods to assess dietary intake (e.g., multiple 24 H-DR and dietary records), making it possible to obtain accurate estimates of dietary intakes. Furthermore, factor analysis (based on principal component-factors) has been considered an interesting approach from the nutritional epidemiology standpoint [56]. Finally, both studies included a large pool of socioeconomic indicators that were used in the current analysis.

The limitations of the study should be taken into account. We used two cross-sectional studies conducted with different objectives (i.e., HELENA study aimed to obtain data concerning: foods consumption and nutrient status, obesity prevalence, dislipidemia, insulin resistance, immunological markers, physical activity, and genetics, whereas the HBS aimed to provide information on the composition of household budgets, the allocation of expenditures, and income distribution). Differences in food habits and culinary use between Europe and Brazil does not allow for establishing the same food groups; however, in order to make then as comparable as possible, we used the same nutritional composition criteria, but including in each region the foods that were usually consumed. Concerning socioeconomic variables, both studies had one in common (number of persons living in the same residence), some similar (education of the mother, FAS in Europe, and total family income in Brazil, geographical region), and some only available in Europe (parental occupation, education of the father, living with the parents, having siblings); these differences prevent us to investigate perfectly equivalent DP associations in both Europe and Brazil.

## 5. Conclusions

We identified similar DP in European and Brazilian adolescents. From a set of socioeconomic and demographic variables, we identified consistent associations between age, education of the mother, parental occupation level, SES, geographical regions (Southern vs. Northern), and Western, Breakfast and Traditional DP in adolescents living in Europe and Brazil. Gender and age of participants resulted in significant differences in DP, illustrating the importance of considering these variables in studies describing DP. Identifying the relation between DP and the socioeconomic factors in adolescents from developed and developing countries is important for understanding food related practices in these populations. This is crucial because adolescence is a transition period, and their food related habits, healthy or not, will be kept through adulthood [15]. Given the various associations between DP and socioeconomic socioeconomic indicators, these findings highlight the relevance of performing a complete assessment of the socioeconomic situation in epidemiological studies in adolescents. These results may support the development of public policies on food and nutrition for adolescents, targeting specific socioeconomic population groups.

## Figures and Tables

**Table 1:** Differences in sociodemographic variables among adolescents participating in the HELENA-Europe 2006/2007 and HBS-Brazil 2008/2009 studies stratified by gender.

	HELENA-EUROPE					HBS-BRAZIL				
Variables	Boys (*n* = 1021)		Girls (*n* = 1192)		*p* Value ^a^	Boys (*n* = 1635)		Girls (*n* = 1559)		*p* Value ^a^
Age (years)–mean (95% CI)	14.9 (14.9, 15.1)		14.9 (14.9, 15.1)		0.230	14.6 (14.4, 14.7)		14.6 (14.5, 15.0)		0.264
	***n***	**%**	***n***	**%**		***n***	**%**	***n***	**%**	
Age categories					0.550					0.127
12.5–13.9	308	43.9	393	56.1		478	49.9	480	50.1	
14–14.9	261	47.4	290	52.6		363	52.5	328	47.5	
15–15.9	239	46.7	273	53.3		290	48.0	314	52.0	
16–17.5	213	47.4	236	52.6		504	53.6	437	46.4	
Socioeconomic status *					0.160					0.07
Low	87	40.1	130	59.9		961	52.8	859	47.2	
Medium	573	46.4	661	53.6		431	48.4	460	51.6	
High	361	47.4	401	52.6		243	50.3	240	49.7	
Number of persons living in a household					0.150					0.657
2–3	293	47.1	329	52.9		233	49.2	241	50.8	
4	442	47.3	492	52.7		472	52.1	433	47.9	
5	186	43.3	244	56.7		378	50.3	374	49.7	
>6	100	44.0	127	55.9		552	51.9	511	48.1	
Geographical region					0.570					0.363
Northern	685	46.7	782	53.3		1067	51.6	1001	48.4	
Southern	336	45.0	410	55.0		568	50.4	558	49.6	
Education mother					0.720					0.590
Low	347	47.3	387	52.7		616	52.9	548	47.1	
High secondary	300	44.2	379	55.8		814	49.1	844	50.9	
University degree	374	46.7	426	53.2		188	54.5	157	45.5	
Education father					0.331					-
Low	340	44.5	424	55.5		-	-	-	-	
High secondary	271	47.1	304	52.9		-	-	-	-	
University degree	383	48.2	412	51.8		-	-	-	-	
Occupation level mother					0.280					-
Low	145	44.6	180	55.4		-	-	-	-	
Medium	393	44.2	497	55.8		-	-	-	-	
High	198	49.2	204	50.7		-	-	-	-	
Undefined class	300	47.6	330	52.4		-	-	-	-	
Occupation level father					0.017					-
Low	223	40.8	323	59.1		-	-	-	-	
Médium	310	47.1	348	52.9		-	-	-	-	
High	316	49.3	325	50.7		-	-	-	-	
Undefined class	166	49.3	171	50.7		-	-	-	-	
Nº siblings **					0.667					-
0	183	47.7	201	52.3		-	-	-	-	
1	513	46.8	583	53.2		-	-	-	-	
>2	348	45.1	423	54.9		-	-	-	-	
Living with parents					0.742					-
Yes	772	46.4	892	53.6		-	-	-	-	
No	284	47.2	318	52.8		-	-	-	-	

^a^ Gender differences using Person test for categorical variables and *t*-test for continuous variables. - Information about these variables was not available in HBS-Brazil. Abbreviations: 95% CI (confidence interval), HELENA; Health, Lifestyle in Europe by Nutrition in Adolescence, HBS; Household Budget Survey. * Based upon Family Affluence Scale (FAS) in HELENA versus total family income in the Brazilian study. ** Number of brothers and sisters.

**Table 2:** Factor loadings of food groups present in the dietary patterns identified among adolescents from HELENA-Europe 2006/2007 study.

	HELENA-EUROPE						
Food Groups	Boys			Girls			
	Western	Traditional European	Breakfast	Breakfast	Western	Traditional European	Monotonous
Bread and rolls	**0.53**	**0.36**	0.12	**0.54**	0.15	0.11	**0.30**
Breakfast cereals	0.17	−0.12	**0.53**	**0.36**	**−0.35**	0.05	−0.23
Cereals	0.06	**0.49**	−0.11	−0.02	−0.05	0.11	**0.69**
Sweet bakery products	−0.06	0.27	0.09	0.00	**0.33**	0.23	−0.10
Savory snacks	**0.39**	0.07	−0.19	−0.02	**0.52**	0.11	0.00
Sugar, honey, syrup	0.29	0.04	0.21	**0.41**	0.03	0.21	−0.05
Non-chocolate confectionery	**0.41**	−0.03	−0.04	0.26	**0.33**	−0.02	−0.16
Chocolate	**0.43**	−0.05	−0.03	0.16	**0.42**	−0.02	−0.03
Vegetable oils, nuts, seeds	−0.04	**0.75**	−0.09	−0.05	−0.03	**0.67**	0.17
Butter, animal fats and margarine	**0.54**	0.01	0.23	**0.64**	−0.02	−0.14	0.10
Sauces	**0.37**	−0.01	−0.07	0.28	0.12	−0.25	0.24
Pulses	−0.21	**0.36**	0.07	−0.10	−0.13	**0.33**	−0.15
Vegetables	0.05	**0.61**	0.19	0.12	−0.03	**0.53**	0.20
Starchy roots and potatoes	0.19	−0.25	0.24	0.23	0.10	−0.13	−0.24
Fruits	0.02	0.17	**0.45**	**0.39**	−0.15	0.18	−0.22
Soups and bouillon	−0.08	−0.01	0.25	−0.04	0.20	0.16	**−0.31**
Coffee and tea	**0.30**	0.07	−0.18	**0.37**	0.12	0.14	−0.05
Fruit and vegetable juices	0.20	−0.01	0.13	0.15	0.09	−0.09	0.00
Sugar-sweetened beverages	**0.51**	−0.08	**−0.38**	0.23	**0.54**	−0.12	−0.06
Alcoholic beverages	0.17	0.05	−0.25	−0.08	**0.32**	0.09	−0.07
Meat, chicken, sausages and ham	0.18	0.18	0.12	0.15	0.11	−0.10	0.16
Fish	−0.21	0.14	0.24	−0.10	−0.17	0.27	−0.13
Eggs	0.00	0.17	0.18	0.08	0.07	**0.49**	−0.11
Milk	−0.07	−0.02	**0.68**	0.28	**−0.49**	0.13	−0.10
Dairy products	0.10	−0.15	**0.34**	0.27	−0.14	−0.17	−0.23
Cheese	**0.30**	**0.52**	−0.08	0.21	0.06	0.10	**0.59**
Other milk products	0.16	0.02	0.01	0.20	0.17	−0.08	−0.15
Mixed products	−0.04	0.04	0.15	0.01	0.03	0.16	0.02
Variance explained (%) in each factor	7.3	7.2	6.4	6.8	5.5	5.7	5.4
Total variance explained (%)	20.9			24.0			

Factors loadings >0.30 positive and negative are shown in bold. Abbreviations: HELENA (Health Lifestyle in Europe by Nutrition in Adolescence).

**Table 3:** Factor loadings of food groups present in the dietary patterns identified among adolescents from HBS-Brazil 2008/2009 study.

HBS-BRAZIL								
Food Groups	Boys				Girls			
	Traditional Brazilian	Western	Snacks	Healthy	Western	Breakfast	Sweets and Fried Foods	Traditional Brazilian
Bread and rolls	**0.63**	0.01	0.11	−0.07	0.09	**0.71**	−0.02	0.05
Breakfast cereals	−0.10	−0.12	0.05	−0.23	0.01	−0.11	**0.58**	0.01
Cereals	**0.38**	0.09	−0.25	−0.07	−0.10	0.14	−0.01	**0.54**
Sweet bakery products	0.07	0.18	**0.50**	0.28	**0.52**	0.12	0.26	−0.09
Savory snacks	0.01	**0.66**	0.01	−0.05	**0.51**	−0.18	−0.05	0.04
Sugar, honey, syrup	−0.03	0.00	0.06	0.02	0.17	**0.32**	0.06	−0.23
Non-chocolate confectionery	−0.10	**0.36**	−0.22	0.09	**0.37**	0.12	0.03	0.08
Chocolate	−0.13	**0.31**	0.12	−0.10	0.27	−0.02	−0.05	0.15
Vegetable oils, nuts, seeds	0.01	0.00	−0.05	0.19	0.13	0.00	**0.66**	−0.03
Butter, animal fats and margarine	**0.66**	0.00	−0.02	0.09	0.05	**0.64**	−0.15	0.12
Sauces	0.02	−0.13	**0.54**	−0.01	0.11	−0.08	−0.21	0.10
Pulses	**0.51**	−0.13	0.10	−0.11	−0.20	0.19	0.01	**0.42**
Vegetables	0.25	0.07	0.24	−0.20	0.10	0.18	−0.02	0.22
Starchy roots and potatoes	0.03	−0.04	0.12	**0.41**	−0.08	−0.09	−0.05	**0.50**
Fruits	0.09	0.07	0.10	**0.44**	0.13	**−0.31**	−0.10	**0.34**
Soups and bouillon	0.14	−0.05	0.20	0.16	−0.05	−0.01	0.00	0.13
Coffee and tea	**0.44**	**−0.38**	**−0.30**	0.18	−0.27	**0.43**	0.08	−0.01
Fruit and vegetable juices	**0.32**	0.28	0.29	0.00	**0.33**	0.18	0.11	**0.48**
Sugar-sweetened beverages	−0.03	**0.74**	0.00	0.00	**0.62**	0.09	−0.01	−0.09
Alcoholic beverages	−0.06	−0.03	0.10	0.14	0.04	−0.11	−0.08	0.01
Meat, chicken, sausages and ham	**0.30**	0.26	−0.06	**0.34**	−0.10	0.07	0.01	**0.35**
Fish	−0.05	−0.10	−0.07	**0.65**	−0.06	−0.06	**0.64**	0.03
Eggs	0.25	−0.14	−0.05	0.01	−0.12	0.18	0.01	−0.06
Milk	0.01	0.00	**0.54**	−0.16	0.11	0.04	−0.20	−0.05
Dairy products	−0.10	0.07	**0.40**	−0.01	0.24	−0.21	−0.26	0.08
Cheese	0.24	0.25	−0.10	−0.24	**0.38**	0.24	−0.07	0.02
Other milk products	−0.01	0.16	0.14	0.14	0.27	**−0.30**	0.06	0.07
Mixed products	−0.04	0.10	−0.16	0.02	0.08	0.09	−0.13	−0.27
Variance explained (%) in each factor	6.8	6.2	5.4	4.8	6.4	6.3	5.4	5.2
Total variance explained (%)	23.2				23.3			

Factors loadings >0.30 positive and negative are shown in bold. Abbreviations: HBS (Household Budget Survey).

**Table 4:** Gender-specific regression β-coefficients (95% IC) between indicators of socioeconomic status and mean scores of DP in adolescents participating in the HELENA-Europe 2006/2007 study.

	HELENA-EUROPE						
	BOYS			GIRLS			
Socioeconomic Variables	*Western*	*Traditional European*	*Breakfast*	*Breakfast*	*Western*	*Traditional European*	*Monotonous*
	β (95% CI)	β (95% CI)	β (95% CI)	β (95% CI)	β (95% CI)	β (95% CI)	β (95% CI)
Age categories							
12.5–13.9	1	1	1	1	1	1	1
14–14.9	0.12 (−0.04, 0.28)	0.51 (−0.10, 0.26)	−0.09 (−0.25, 0.10)	−0.05 (−0.20, 0.10)	−0.07 (−0.23, 0.07)	0.09 (−0.06, 0.24)	−0.15 (−0.30, −0.00)
15–15.9	**0.44 (0.28, 0.61)**	0.16 (−0.01, 0.32)	−0.21 (−0.38, 0.04)	**0.28 (0.12, 0.43)**	0.07 (−0.08, 0.22)	0.03 (−0.12, 0.18)	−0.16 (−0.31, −0.00)
16–17.5	**0.45 (0.28, 0.62)**	**0.31 (0.13, 0.48)**	**−0.32 (−0.50, −0.15)**	**0.22 (0.06, 0.38)**	0.07 (−0.09, 0.23)	0.12 (−0.04, 0.28)	**−0.32 (−0.48, −0.16)**
Socioeconomic status *							
Low	1	1	1	1	1	1	1
Medium	0.15 (−0.07, 0.38)	−0.24 (−0.46, −0.01)	**0.29 (0.06, 0.51)**	**0.32 (0.13, 0.50)**	0.03 (−0.16, 0.22)	−0.06 (−0.25, 0.12)	0.12 (−0.07, 0.31)
High	**0.43 (0.20, 0.66)**	**−0.40 (−0.63, −0.17)**	0.39 (0.16, −0.10)	**0.55 (0.35, 0.74)**	−0.07 (−0.27, 0.13)	**−0.33 (−0.53, −0.14)**	0.25 (0.05, 0.45)
Number of persons living in a household							
2–3	1	1	1	1	1	1	1
4	0.01 (−0.14, 0.16)	−0.00 (−0.15, 0.14)	0.07 (−0.08, 0.21)	0.14 (−0.00, 0.28)	**−0.18 (−0.32, −0.04)**	0.02 (−0.12, 0.16)	0.03 (−0.11, 0.17)
5	0.11 (−0.07, 0.29)	−0.07 (−0.25, 0.12)	**0.28 (0.10, 0.46)**	0.05 (−0.11, 0.22)	−0.06 (−0.22, 0.11)	−0.11 (−0.27, 0.05)	0.07 (−0.09, 0.24)
>6	0.14 (−0.08, 0.37)	−0.10 (−0.33, 0.13)	**0.28 (0.06, 0.51)**	0.17 (−0.03, 0.37)	0.04 (−0.17, 0.24)	**−0.28 (−0.49, −0.08)**	0.17 (−0.03, 0.38)
Geographical region							
Northern	1	1	1	1	1	1	1
Southern	**−0.92 (−1.03, −0.82)**	**0.92 (0.78, 1.06)**	0.05 (−0.07, 0.18)	**−0.81 (−0.91, −0.70)**	**−0.30 (−0.41, −0,19)**	**0.87 (0.76, 0.99)**	**−0.40 (−0.53, −0.27)**
Education mother							
Low	1	1	1	1	1	1	1
High secondary	**−0.21 (−0.36, −0.05)**	**0.21 (0.05, 0.36)**	0.14 (−0.01, 0.29)	−0.05 (−0.19, 0.09)	−0.12 (−0.26, 0.02)	**0.19 (0.05, 0.33)**	−0.06 (−0.21, 0.07)
University degree	**−0.15 (−0.30, −0.01)**	0.06 (−0.08, 0.21)	**0.37 (0.22, 0.51)**	0.09 (−0.05, 0.23)	**−0.43 (−0.56, −0.29)**	0.12 (−0.02, 0.25)	0.12 (−0.02, 0.26)
Education father							
Low	1	1	1	1	1	1	1
Higher secondary	−0.16 (−0.32, 0.00)	**0.30 (0.14, 0.46)**	−0.01 (−0.17, 0.14)	−0.09 (−0.24, 0.06)	−0.04 (−0.19, 0.11)	**0.22 (0.07, 0.37)**	−0.02 (−0.17, 0.12)
University degree	−0.06 (−0.21, 0.09)	0.11 (−0.04, 0.25)	**0.37 (0.29, 0.52)**	0.07 (−0.07, 0.20)	**−0.33 (−0.46, −0.19)**	0.07 (−0.07, 0.20)	0.07 (−0.07, 0.20)
Occupation level mother							
Low	1	1	1	1	1	1	1
Medium	0.08 (−0.06, 0.22)	−0.14 (−0.28, −0.00)	**0.18 (0.04, 0.31)**	**0.23 (0.10, 0.35)**	−0.00 (−0.13, 0.12)	−0.01 (−0.14, 0.11)	−0.00 (−0.13, 0.12)
High	−0.01 (−0.18, 0.16)	−0.16 (−0.33, −0.00)	**0.31 (0.14, 0.48)**	**0.25 (0.08, 0.41)**	**−0.21 (−0.38, −0.05)**	**−0.24 (−0.40, −0.07)**	**0.26 (0.10, 0.43)**
Occupation level father							
Low	1	1	1	1	1	1	1
Medium	−0.06 (−0.21, 0.09)	0.20 (−0.33, −0.00)	−0.02 (−0.17, 0.13)	**0.19 (0.05, 0.32)**	**−0.16 (−0.30, −0.02)**	**0.19 (0.05, 0.33)**	−0.02 (−0.16, 0.12)
High	0.13 (−0.01, 0.28)	−0.00 (−0.15, 0.14)	**0.21 (0.06, 0.36)**	**0.25 (0.11, 0.39)**	**−0.20 (−0.34, −0.05)**	−0.09 (−0.23, 0.06)	0.12 (−0.02, 0.26)
Nº siblings **							
0	1	1	1	1	1	1	1
1	−0.06 (−0.23, 0.10)	−0.02 (−0.19, 0.14)	0.05 (−0.11, 0.21)	0.15 (0.00, 0.31)	**−0.28 (−0.43, −0.12)**	0.08 (−0.07, 0.24)	−0.07 (−0.22, 0.09)
>2	0.07 (−0.10, 0.25)	−0.12 (−0.30, 0.06)	**0.26 (0.08, 0.03)**	0.17 (0.01, 0.33)	−0.12 (−0.28, 0.04)	−0.10 (−0.27, 0.06)	0.07 (−0.09, 0.23)
Living with Parents							
Yes	1	1	1	1	1	1	1
No	0.03 (−0.11, 0.17)	−0.06 (−0.20, 0.08)	**−0.26 (−0.40, −0.12)**	**0.17 (0.04, 0.30)**	0.04 (−0.09, 0.17)	0.03 (−0.10, 0.16)	−0.00 (−0.14, 012)

β (95% CI) value with significant associations (*p* < 0.05) are shown in bold. 1 = Represents the reference category. Abbreviations: 95% CI (confidence interval), HELENA (Health Lifestyle in Europe by Nutrition in Adolescence), * Based upon Family Affluence Scale (FAS). ** Number of brothers and sisters.

**Table 5:** Gender specific regression β-coefficients (95% IC) between indicators of socioeconomic status and mean scores of DP in adolescents participating in HBS-Brazil 2008/2009 studty.

	HBS-BRAZIL							
	BOYS				GIRLS			
Socioeconomic Variables	*Traditional Brazilian*	*Western*	*Snacks*	*Healthy*	*Western*	*Breakfast*	*Sweets and Fried Foods*	*Traditional Brazilian*
	β (95% CI)	β (95% CI)	β (95% CI)	β (95% CI)	β (95% CI)	β (95% CI)	β (95% CI)	β (95% CI)
Age categories								
12.5–13.9	1	1	1	1	1	1		1
14–14.9	−0.04 (−0.17, 0.10)	−0.00 (−0.14, 0.13)	−0.00 (−0.11, 0.11)	−0.04 (−0.18, 0.10)	0.07 (−0.15, 0.28)	0.20 (−0.05, 0.44)	**−0.04 (0.16, 0.10)**	0.10 (−0.19, 0.39)
15–15.9	0.14 (−0.00, 0.29)	0.03 (−0.12, 0.18)	−0.05 (−0.16, 0.07)	0.05 (−0.10, 0.20)	0.07 (−0.17, 0.32)	0.11 (−0.15, 0.37)	0.05 (−0.09, 0.19)	−0.15 (−0.41, 0.11)
16–17.5	**0.32 (0.19, 0.44)**	−0.06 (−0.19, 0.06)	−0.05 (−0.15, 0.05)	−0.07 (−0.20, 0.05)	0.06 (−0.13, 0.25)	0.10 (−0.15, 0.34)	0.16 (−0.03, 0.36)	0.14 (−0.10, 0.38)
Socioeconomic status *								
Low	1	1	1	1	1	1	1	1
Medium	**0.18 (0.07, 0.30)**	**0.63 (0.53, 0.73)**	0.04 (−0.05, 0.13)	0.07 (−0.05, 0.18)	**0.44 (0.27, 0.62)**	0.01 (−0.19, 0.22)	0.15 (−0.01, 0.31)	0.03 (−0.17, 0.24)
High	0.12 (−0.02, 0.26)	**1.20 (1.07, 1.32)**	0.08 (−0.03, 0.19)	0.01 (−0.13, 0.15)	**0.87 (0.66, 1.07)**	−0.10 (−0.35, 0.15)	−0.08 (−0.23, 0.07)	**−0.28 (−0.52, −0.04)**
Number of persons living in a household								
2 to 3	1	1	1	1	1	1	1	1
4	0.11 (−0.05, 0.26)	−0.03 (−0.19, 0.12)	0.01 (−0.12, 0.13)	−0.03 (−0.19, 0.12)	−0.12 (−0.37, 0.13)	−0.15 (−0.43, 0.12)	0.17 (−0.02, 0.36)	−0.02 (−0.31, 0.27)
5	0.08 (−0.09, 0.25)	**−0.18 (−0.35, −0.02)**	−0.02 (−0.15, 0.11)	−0.09 (−0.26, 0.07)	−0.23 (−0.46, 0.01)	0.18 (−0.10, 0.46)	**0.25 (0.08, 0.42)**	0.07 (−0.19, 0.34)
>6	−0.04 (−0.19, 0.11)	**−0.52 (−0.67, −0.37)**	0.02 (−0.10, 0.14)	0.02 (−0.14, 0.17)	**−0.50 (−0.75, −0.24)**	0.12 (−0.18, 0.42)	−0.02 (−0.17, 0.12)	−0.03 (−0.32, 0.26)
Geographical region								
Northern	1	1	1	1	1	1	1	1
Southern	0.13 (−0.01, 0.28)	**0.49 (0.34, 0.63)**	0.04 (−0.08, 0.16)	**−0.26 (−0.40, −0.11)**	**0.29 (0.15, 0.44)**	−0.14 (−0.30, 0.02)	**−0.22 (−0.34, −0.09)**	**0.19 (0.03, 0.35)**
Education mother								
Low	1	1	1	1	1	1	1	1
High secondary	**0.20 (0.10, 0.31)**	**0.37 (0.27, 0.47)**	0.07 (−0.01, 0.15)	0.08 (−0.03, 0.18)	**0.38 (0.22, 0.54)**	0.07 (−0.11, 0.25)	−0.07 (−0.23, 0.09)	0.05 (−0.15, 0.24)
University degree	**0.22 (0.06, 0.38)**	**0.97 (0.81, 1.13)**	**0.14 (0.01, 0.27)**	0.16 (−0.00, 0.33)	**0.79 (0.53, 1.05)**	−0.09 (−0.46, 0.27)	−0.07 (−0.29, 0.15)	−0.08 (−0.35, 0.18)

β (95% CI) value with significant associations (*p* < 0.05) are shown in bold. 1 = Represents the reference category. Abbreviations: Abbreviations: 95% CI (confidence interval), HBS (Household Budget Survey). * Based upon total family income in the Brazilian study.

## Supplementary Materials

Supplementary File 1

## Abbreviations

CI	95% confidence interval
DP	dietary patterns
FAS	Family Affluence Scale
HBS	Household Budget Survey (Brazil)
HELENA-DIAT	HELENA-Dietary Assessment Tool
HELENA-study	Healthy Lifestyle in Europe by Nutrition in Adolescence
IBGE	Brazilian Institute of Geography and Statistics
SES	socioeconomic status
YANA-C	Young Adolescents’ Nutrition Assessment software
24 H-DR	24-h dietary recalls

## Appendix A

**Table A1:** Description of the foods that composed each of the 28 food groups included in the factor analysis (HELENA-Europe 2006/2007 and HBS-Brazil 2008/2009).

	HELENA-EUROPE	HBS-BRAZIL
Food Groups	Composition	Composition
Bread and bread rolls	Sliced bread, whole bread, white bread, rolls bread, crispbread, rusks	Salt bread, whole bread
Breakfast cereals	Breakfast cereals, oatmeal	Breakfast cereals, oatmeal, porridge
Cereals	Flour, pasta, rice and other cereals	Flour, pasta, rice, rice preparations, whole rice, corn, corn recipes,
Sweet bakery products	Cakes, pies, biscuits, croissants, brioches	Cakes, pies, biscuits, sweet breads diet and light, cakes diet and light
Savory snacks	Crisps, salty biscuits, aperitif biscuits	Salty biscuits, pizza
Sugar, honey, syrup	Table sugar, honey, syrup, jam, dessert sauces (excluding chocolate sauce), water ice, sorbet (excluding ice cream)	Table sugar, honey, syrup, jam, brown sugar, candy in sugar syrup
Non-chocolate confectionery	All confectionary non chocolate, candies	All confectionary non chocolate, candies
Chocolate	Chocolate, candy bars, chocolate paste, chocolate confetti/flakes, chocolate sauces	Chocolate, candy bars, chocolate paste, chocolate confetti/flakes, chocolate sauces, cocoa powder
Vegetable oils, nuts, seeds	Vegetable oils (olive oil, soya oil, corn oil, canola oil) olives, avocado, nuts, seed spreads	Vegetable oils (olive oil, soya oil, corn oil, canola oil) olives, avocado, nuts, seed spreads
Butter, animal fats and margarine	Butter, margarine and lipids of mixed origins, animal fats	Butter, margarine and lipids of mixed origins, animal fats
Sauces	Mayonnaises and similar, dressing sauces, gravy, tomato sauces, other sauces (excluding dessert sauces)	Mayonnaises and similar, dressing sauces, tomato sauces, other sauces (excluding dessert sauces), condiments (ketchup, mustard)
Pulses	All types of beans, lentils, chickpeas (others excluding fresh peas, sweet corn and broad bean)	All types of beans, lentils, chickpeas, peas
Vegetables excluding potatoes	All the vegetables excluding potatoes	All the vegetables excluding potatoes
Starch roots and potatoes	Starch roots, potatoes	Starch roots , potatoes, sweet potatoes, manioc
Fruits	All fresh fruits	All fresh fruits
Soups and bouillon	Soups, bouillon	Soups, bouillon
Coffee and tea	Coffee and tea	Coffee and tea
Fruit and vegetable juices	Fruit and vegetable juices	Fruit and vegetable juices
Sugar-sweetened beverages	Carbonated, soft, isotonic drinks, including not alcoholic wine, not alcoholic beer	Carbonated, soft, isotonic drinks, including not alcoholic wine, not alcoholic beer
Alcoholic beverages	Beer, wine, cider, other alcoholic beverages	Beer, brandy, wine, cider, other alcoholic beverages
Meat, chicken, sausages and ham	Cow meat, pork meat, chicken, sausages and ham, beef, veal, mutton/lamb, goat	Cow meat, pork meat, chicken, sausages and ham, beef, canned meat, hamburgers
Fish	Fish, crustaceans, mollusks, fish mousse, fish pate, tarama	Fish, crustaceans, mollusks, fish, canned fish, salt fish
Eggs	Eggs, omelets	Eggs, omelets
Milk	Milk, white milk, buttermilk	Milk, white milk, whole milk
Dairy products	Yogurt, fromage blanc, yogurt beverages, chocolate milk, probiotic beverages	Yogurt, fromage blanc, yogurt beverages, probiotic beverages
Cheese	All cheese excluding fromage blanc (quark)	All cheese excluding fromage blanc (quark)
Other milk products	Desserts and puddings milk based (including ice cream), flan, mousse, tiramisu, creams (including non-dairy and coffee creams)	Desserts and puddings milk based (including ice cream), milkshakes with fruits
Mixed products	Meat substitutes, vegetarian products, vegetarian burgers, tempeh, tofu, spring roll, products for special nutritional use, other miscellaneous	Lasagna ready for consumption, yakisoba, other miscellaneous, Japanese food, take way foods.
