# Supplementary material for: Dietary Patterns in European and Brazilian Adolescents: Comparisons and Associations with Socioeconomic Factors
Source: Nutrients. 2018 Jan 9;10(1):57. doi: 10.3390/nu10010057 (PMC5793285; doi:10.3390/nu10010057)
Supplement: Supplementary File 1 [file nutrients-10-00057-s001.pdf]

Additional File 1. Description of the foods that composed each of the 28 food groups included in the factor analysis (HELENA-Europe 2006/2007 and HBS-Brazil 2008/2009).

	HELENA - EUROPE	HBS- BRAZIL
**Food Groups**	**Composition**	**Composition**
**Bread and bread rolls**	Sliced bread, whole bread, white bread, rolls bread, crispbread, rusks	Salt bread, whole bread
**Breakfast cereals**	Breakfast cereals, oatmeal	Breakfast cereals, oatmeal, porridge
**Cereals**	Flour, pasta, rice and other cereals	Flour, pasta, rice, rice preparations, whole rice, corn, corn recipes,
**Sweet bakery products**	Cakes, pies, biscuits, croissants, brioches	Cakes, pies, biscuits, sweet breads diet and light, cakes diet and light
**Savory snacks**	Crisps, salty biscuits, aperitif biscuits	Salty biscuits, pizza
**Sugar, honey, syrup**	Table sugar, honey, syrup, jam, dessert sauces (excluding chocolate sauce), water ice, sorbet (excluding ice cream)	Table sugar, honey, syrup, jam, brown sugar, candy in sugar syrup
**Confectionary non chocolate**	All confectionary non chocolate, candies	All confectionary non chocolate, candies
**Chocolate**	Chocolate, candy bars, chocolate paste, chocolate confetti/flakes, chocolate sauces	Chocolate, candy bars, chocolate paste, chocolate confetti/flakes, chocolate sauces, cocoa powder
**Vegetable oils, nuts, seeds**	Vegetable oils (olive oil, soya oil, corn oil, canola oil) olives, avocado, nuts, seed spreads	Vegetable oils (olive oil, soya oil, corn oil, canola oil) olives, avocado, nuts, seed spreads
**Butter, animal fats and margarine**	Butter, margarine and lipids of mixed origins, animal fats	Butter, margarine and lipids of mixed origins, animal fats
**Sauces**	Mayonnaises and similar, dressing sauces, gravy, tomato sauces, other sauces (excluding dessert sauces)	Mayonnaises and similar, dressing sauces, tomato sauces, other sauces (excluding dessert sauces), condiments (ketchup, mustard)
**Pulses**	All types of beans, lentils, chickpeas (others excluding fresh peas, sweet corn and broad bean)	All types of beans, lentils, chickpeas, peas
**Vegetables excluding potatoes**	All the vegetables excluding potatoes	All the vegetables excluding potatoes
**Starch roots and potatoes**	Starch roots, potatoes	Starch roots , potatoes, sweet potatoes, manioc
**Fruits**	All fresh fruits	All fresh fruits
**Soups and bouillon**	Soups, bouillon	Soups, bouillon
**Coffee and tea**	Coffee and tea	Coffee and tea
**Fruit and vegetable juices**	Fruit and vegetable juices	Fruit and vegetable juices
**Sugar sweetened beverages**	Carbonated, soft, isotonic drinks, including not alcoholic wine, not alcoholic beer	Carbonated, soft, isotonic drinks, including not alcoholic wine, not alcoholic beer
**Alcoholic beverages**	Beer, wine, cider, other alcoholic beverages	Beer, brandy, wine, cider, other alcoholic beverages
**Meat, chicken, sausages and ham**	Cow meat, pork meat, chicken, sausages and ham, beef, veal, mutton/lamb, goat	Cow meat, pork meat, chicken, sausages and ham, beef, canned meat, hamburgers
**Fish**	Fish, crustaceans, mollusks, fish mousse, fish pate, tarama	Fish, crustaceans, mollusks, fish, canned fish, salt fish
**Eggs**	Eggs, omelets	Eggs, omelets
**Milk**	Milk, white milk, buttermilk	Milk, white milk, whole milk
**Dairy products**	Yogurt, fromage blanc, yogurt	Yogurt, fromage blanc, yogurt beverages,

	beverages, chocolate milk, probiotic beverages	probiotic beverages
**Cheese**	All cheese excluding fromage blanc (quark)	All cheese excluding fromage blanc (quark)
**Other milk products**	Desserts and puddings milk based (including ice cream), flan, mousse, tiramisu, creams (including non-dairy and coffee creams)	Desserts and puddings milk based (including ice cream), milkshakes with fruits
**Mixed products**	Meat substitutes, vegetarian products, vegetarian burgers, tempeh, tofu, spring roll, products for special nutritional use, other miscellaneous	Lasagna ready for consumption, yakisoba, other miscellaneous, Japanese food, take way foods.
